# Long-Term Follow-Up following Condylotomy in a Case of Traumatic Unilateral Anterosuperior Mandibular Condyle Dislocation

**DOI:** 10.1155/2019/6810461

**Published:** 2019-05-14

**Authors:** Syed Nabil, Elavarasi Kuppusamy, Rifqah Nordin, Abdul Jabar Nazimi, Roszalina Ramli

**Affiliations:** Oral and Maxillofacial Surgery, Faculty of Dentistry, The National University of Malaysia, Jalan Raja Muda Abdul Aziz, 50300 Kuala Lumpur, Malaysia

## Abstract

Anterosuperior temporomandibular joint dislocation is rare. Manual reduction of such dislocation is difficult especially when treatment is delayed. Therefore, it has an increased likelihood of needing surgical intervention to achieve reduction. The authors report a case of an anterosuperior temporomandibular dislocation in a 19-year-old male following a motor vehicle accident. Difficulties were encountered in reducing the dislocation necessitating surgically assisted reduction. The long-term outcome following management by condylotomy is reported. This present report also reviews the literature regarding the classification and management of this uncommon dislocation.

## 1. Introduction

Temporomandibular joint dislocation (TMJD) is an abnormal condition in which the head of the condyle displaces from its usual position in the glenoid fossa in the squamotemporal portion of the cranial base [1]. TMJD can involve only one or bilateral joints, and it can be dislocated either anteriorly, laterally, medially, posteriorly, or superiorly [1]. The cause of TMJD can either be traumatic or nontraumatic [2]. Nontraumatic dislocations are more common and precipitated by events such as forceful or excessive opening of the mouth during laughing, yawning, and dental or endoscopy treatment, during anesthetic intubation, and during eating or as consequences of seizures [1]. The nontraumatic TMJD is caused by the laxity of the surrounding connective tissue and generally can be managed by conservative reduction [2]. Traumatic TMJD meanwhile has been reported to occur following a fall, road traffic accident, domestic accident, or interpersonal violence [1]. TMJD can also be classified as acute, recurrent/habitual, or long-standing/chronic based on the dislocation duration [3]. Acute dislocation is the most frequent. When acute dislocations are not reduced for more than 72 hours, it can be defined as long-standing dislocations [4]. Recurrent or habitual dislocation occurs in repeated episodes and may become more frequent if left untreated [3].

A subtype of TMJD that is notoriously difficult to reduce is the anterosuperior dislocation (ASD) [5, 6]. ASD is described as a condition where the condyle is displaced anteriorly beyond the articular eminence then superiorly medial to the zygomatic arch to enter into the temporal fossa [6]. This type of dislocation is uncommon with only a handful of cases reported. The authors report a case of ASD in an intact condyle following a motor vehicle accident. Additionally, this article would describe the long-term outcome of such case following management with intraoral condylotomy.

## 2. Case Report

**Figure 1:**
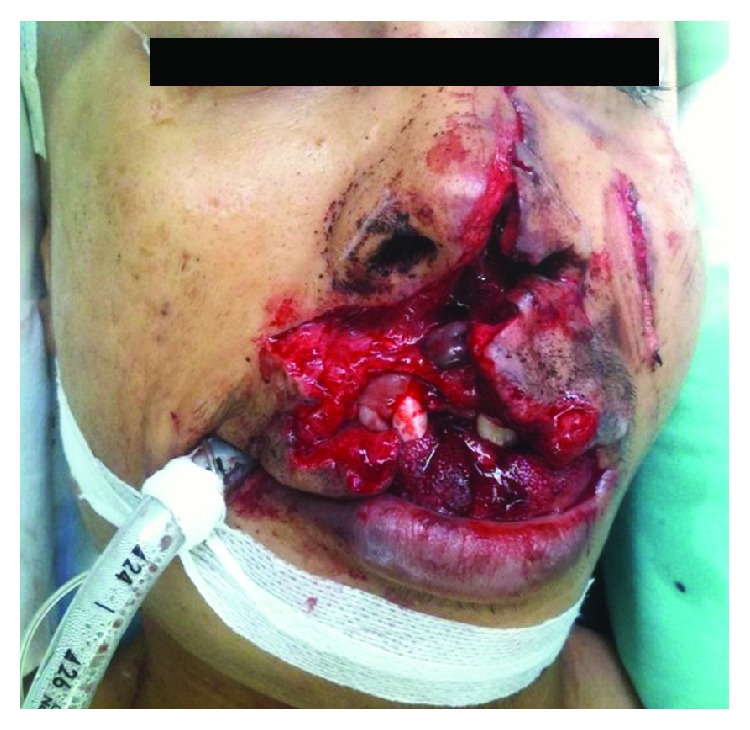
Facial injury sustained.

**Figure 2:**
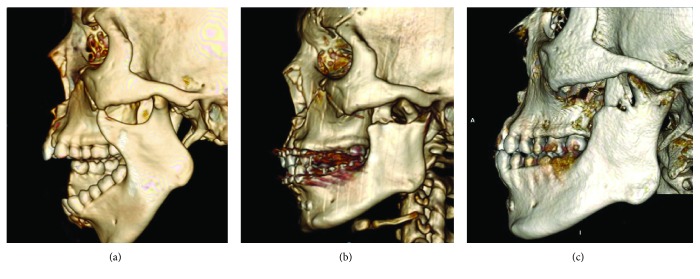
CT scan image of the left temporomandibular joint at (a) day 1 of trauma, (b) two days postsurgery, and (c) one year postsurgery.

A 19-year-old man was brought to the emergency department following a road traffic accident after his motorcycle skidded and hit the road divider. His Glasgow Coma Scale (GSC) on initial examination was 12/15. He sustained laceration of his upper lip and tongue, comminuted fracture of the right mandible parasymphysis, and avulsed teeth 11, 12, 41, 42, 43, and 44 (Figure 1). He was intubated immediately for airway protection. An emergency head CT scan showed that he also sustained depressed fracture of the frontal bone with subdural and epidural hemorrhage. CT scan also showed right parasymphysis mandible fracture and dislocated left condyle (Figure 2(a)). The left condyle was dislocated anteriorly and superiorly into the infratemporal fossa medial to the zygomatic arch. There were no fractures of the condyle and zygomatic arch.

He underwent emergency craniotomy with evacuation of blood clot by the neurosurgical team. In the same setting, the facial laceration injury was sutured and an arch bar with intraosseous wiring was placed to stabilize the fractured mandible. Condyle dislocation reduction was also attempted. Due to the orotracheal intubation tube, the occlusion was not assessed following reduction. The patient was then transferred to the intensive care unit (ICU) subsequently with the orotracheal intubation kept in place. Following extubation 5 days later, it was noted that the patient kept his mouth open without any closure movement. There was also excessive drooling of saliva due to the inability to close his mouth. On examination, his mandible movement appeared restricted and the mandible was unable to move in any direction. He was not obeying instruction well. Multiple manual reduction attempts at bedside were unsuccessful.

An open reduction and internal fixation was planned for the right parasymphysis of mandible fracture, and it was planned to perform reduction of the dislocated condyle on the left side. Owing to the patient's neurological injury, the surgery could only be done 2 weeks after the injuries were sustained. In view of the ASD condyle and the prolonged period of dislocation, we anticipated difficult reduction. This was discussed with the patient and his family, and it was decided if the need for open or surgical reduction arises, they prefer surgical approach to be done intraorally. During the surgery under general anesthesia with muscle relaxation, initial attempts were made to reduce the left condylar dislocation by using manual traction by Hippocratic method and then with the assistance of a mouth gag but proved to be unsuccessful. Our next attempt was to release the intraosseous wiring at the parasymphysis fracture site effectively rendering the mandible in two separate pieces to simulate mandibulotomy-assisted reduction as described by previous clinicians [7, 8]. Once this was not successful, we went ahead with an intraoral condylotomy on the left side by piezoelectric surgery. First, a coronoidectomy was done to get access to the condyle. Then, the condylar neck was osteotomized using the piezosurgery, and the mandible was then able to be pushed back into occlusion. Finally, open reduction and fixation of the right parasymphysis fracture was performed and stable occlusion was achieved.

Postoperative CT scan confirmed the reduction of the dislocation (Figure 2(b)). There was a slight deviation of the jaw to the left and mouth opening was 19 mm. Occlusion was acceptable and elastics were placed for 6 weeks. Jaw exercises were encouraged, and review after 2 months postoperatively showed improvement in mouth opening at 40 mm with stable occlusion. The patient was then referred to a prosthodontist for further rehabilitation and treatment of the missing teeth.

**Figure 3:**
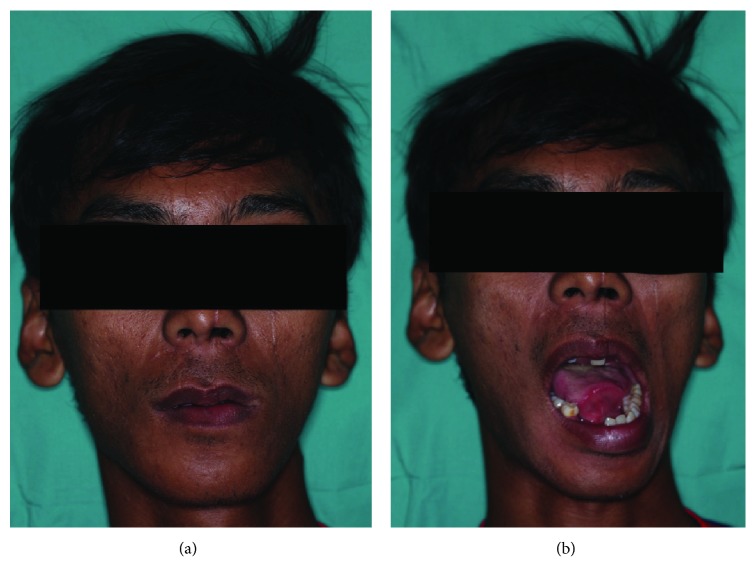
Extraoral presentation on closing and opening of the mouth one year postsurgery.

**Figure 4:**
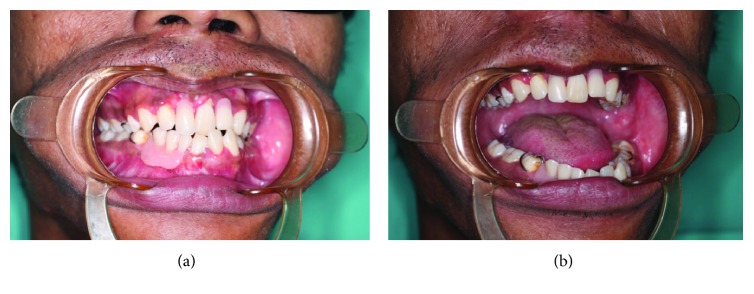
Occlusion on biting and opening of the mouth with dentures one year postsurgery.

On 1-year follow-up, the patient presented with no complaint. Clinically, there was no tenderness at the joint or muscle of mastication on palpation or during movement. However, there was mild asymmetry of the jaw with the chin deviated 2 mm to the left (Figure 3). Mouth opening was maintained at 40 mm with deviation to the left on opening (Figure 4). Occlusion was good with upper and lower dentures in place. CT scan shows union between the condyle head and the condylar process stump (Figure 2(c)). Its position remains as seen immediately after surgery. The stump of the condylar process meanwhile has remodeled to form a neocondyle.

## 3. Discussion

The description of ASD can be rather confusing. Several previous authors used different terms for dislocation of the condyle medial to the zygomatic arch similar to this reported case including superolateral dislocation (SLD) [9], anteromedial dislocation (AMD) [2], and ASD [6]. We describe our case as ASD as the condyle is anterior and superior to its original position in the glenoid fossa. Technically, the terms SLD and AMD for such dislocation are inaccurate as the condyle is not located medial/lateral to the glenoid fossa but instead is medial/lateral to the zygomatic arch. To add further confusion, ASD has been suggested to be included under lateral dislocation classification. Allen and Young first attempted to classify cases of lateral TMJD to types I and II for lateral subluxation and lateral complete dislocation, respectively [10]. Later, Satoh et al. suggested further refinement of lateral TMJD by dividing type II to subtypes IIA, IIB, and IIC [11]. They described unhooked condyle in lateral TMJD (type IIA), laterally hooked condyle above the zygomatic arch (type IIB), and lodgment of the condyle under a fractured zygomatic arch (type IIC) [11]. The next revision of this classification was made by Prabhakar and Singla who described the condyle of an intact mandible being medial to unfractured zygoma [6]. Type III as defined by Prabhakar and Singla is more precisely an anterior-superior dislocation (as described by the authors themselves); thus, together with type IIC from Satoh et al. has deviated from the “lateral” TMJD classification as described initially by Allen and Young [6, 10, 11]. Further refinement was made to complete the type III classification to type IIIA or type IIIB based on the presence or absence of mandibular fracture [5]. Most recently, Rahman et al. analyzed previous classification and suggested a complete classification for lateral TMJD [12]. While the classification is comprehensive, it technically lumps together an anterior vector dislocation (anterosuperior) with a lateral vector dislocation (lateral and superolateral).

Untreated or persistent acute condyle displacement due to failure to diagnose or inadequate treatment rendered for the patient can lead to “long standing” TMJD [3]. Once the condyle becomes abnormally positioned for a period of time, it causes muscle spasm, soft tissue fibrosis, and soft tissue ingrowth into the glenoid fossa [2–4]. Thus, prolonged dislocations will be more difficult to be repositioned [2–4]. Several authors agree with this and further suggest the use of the duration of the dislocation as a guide in managing long-standing TMJD cases [2, 4]. Huang et al. suggested closed reduction in a dislocation of less than 3 weeks, open reduction using wire traction at the angle of mandible for a dislocation persisting more than 4-12 weeks, and more complicated surgical procedures such as condylectomy, condylotomy, myotomy, and TMJ prosthesis for dislocations persisting for more than 6 months [4]. Rattan and Rai meanwhile proposed trial of manual reduction and anterior traction using elastics before proceeding with indirect open reduction with the use of transosseous wires or hooks, direct open reduction via preauricular approach in TMJD that persist for more than one month, and orthognathic surgical procedure in cases of more than 6 months [2]. There is no consensus yet on when should an acute dislocation phase ends and the long-standing starts [3]. Huang et al. define a dislocation which has been left untreated or inadequately treated for more than 72 hours while Rattan et al. suggest a longer period of 1 month [2, 4]. In the presented case, the dislocation was attempted to be reduced at 14 days. The difficulty in manual reduction clearly shows the extent of spasm, fibrosis, and tissue ingrowth to be well advanced. There is also a possibility of a mechanical obstruction with the condyle under the zygomatic arch. Hence, an invasive procedure was eventually needed involving condylotomy and coronoidectomy on the left side as described by Pappachan et al. [13].

In ASD, the need for surgical reduction should be anticipated. From the literature, it is noted that in the nine previously reported cases, 56% (5/9) of the cases with this type of dislocation need open surgery reduction [5]. The remaining 33% (3/9) had assisted manual reduction such as the use of mouth gags [9] and zygomatic bone hook traction [5, 14]. Furthermore, the period of time before reduction in cases of ASD further contributes to the failures in manual reduction [5]. Several surgical modalities have been reported in the management of difficult cases of TMJ dislocation. These include direct reduction via preauricular incision [2], condylectomy [15], condylotomy [13], inverted L-shaped ramus osteotomy [16], bilateral vertical-oblique osteotomy of ramus [17], and midline mandibulotomy [7]. In the reported case, after conservative attempts and using of the right parasymphysis fracture to simulate midline mandibulotomy reduction failed, we proceed to perform condylotomy. Due to the expected shortening of the ramus height, long-term undesirable changes that could possibly develop include the presence of malocclusion, the deviation to the operated side when opening the mouth, loss of lateral excursion, TMJ dysfunction symptoms, and open bite [18, 19]. Clinical examination at 1-year postsurgery showed deviation of the mandible to the left side on jaw opening and the chin was noted to be shifted 2 mm to the left when resting. The patient however gained an excellent range in mouth opening with good occlusion, and normal function of the jaw was restored. Radiographically, although the left condyle head was still at its dislocated position, the stump of the condylar process has remodeled to form a neocondyle allowing satisfactory functioning of the jaw. While this shows acceptable outcome, a long-term effect of a unilateral shortening of the ramus height is still not clear. Previous reported case of condylotomy for anteriorly displaced condyles also reported similar favorable outcome [13, 20].

Condylotomy can be performed transorally, which avoids the cutaneous scar and risk for facial nerve injury. Risks to the other surrounding structures such as maxillary artery meanwhile differ among the different devices used to perform the osteotomy. Osteotomy around the condyle can be performed using piezosurgery, drill, saw, osteotome, and even gigli saw. The use of piezosurgical instruments has the advantage of not damaging the soft tissue and at the same time allowing and improving precision in osteotomy. This allows access to this limited area with minimal damage to the surrounding tissue. The fact that the joint was dislocated anteriorly provides an advantage for the surgeon to access the condyle in the reported case.

In summary, ASD is a rare occurrence of condyle dislocation anterior to the glenoid fossa and should not be classified together with lateral vector dislocations. When ASD is identified, surgical management should be anticipated and planned due to the low likelihood of success for manual reduction especially when treatment provision is delayed. There is no proper evidence to suggest or guide which surgical intervention would provide the best long-term outcome for the management of ASD. Condylotomy especially with piezoelectric surgery is a viable and safe option especially when intraoral approach is preferred.
